# Case Report: Persistent indirect hyperbilirubinemia caused by Gilbert syndrome misdiagnosed as drug-induced liver injury during tuberculosis treatment

**DOI:** 10.3389/fmed.2026.1903970

**Published:** 2026-07-16

**Authors:** Ping Liu, Yuming Xu

**Affiliations:** 1 Department of Internal Medicine, Harbin Chest Hospital of Harbin Medical University, Harbin, China; 2 Department of Operating Room, Harbin Chest Hospital of Harbin Medical University, Harbin, China

**Keywords:** drug-induced liver injury, gilbert syndrome, tuberculosis, UGT1A1, unconjugated hyperbilirubinemia

## Abstract

**Background:**

Drug-induced liver injury (DILI) is a common complication of anti-tuberculosis therapy and frequently leads to treatment interruption or modification. In routine clinical practice, liver function abnormalities during tuberculosis treatment are often attributed to DILI. However, isolated indirect hyperbilirubinemia in the presence of persistently normal transaminase levels represents an atypical biochemical pattern that is inconsistent with classical hepatocellular injury and may indicate alternative underlying conditions, posing a diagnostic challenge in tuberculosis management.

**Case presentation:**

We report the case of a 22-year-old Han Chinese woman treated for smear-negative pulmonary tuberculosis who developed recurrent elevations in total and indirect bilirubin while alanine aminotransferase and aspartate aminotransferase levels remained persistently within normal ranges. These abnormalities were repeatedly misinterpreted as suspected DILI, resulting in multiple interruptions and modifications of anti-tuberculosis regimens. Bilirubin levels continued to fluctuate despite drug withdrawal, suggesting a non-hepatocellular etiology. Repeated treatment interruption contributed to a delay in effective tuberculosis management exceeding 1 year and was associated with radiological disease progression and cavity formation.

**Management and outcome:**

Further evaluation, including *UGT1A1* genetic testing, identified heterozygous promoter (c.-41_-40dupTA) and coding (c.211G>A, p.Gly71Arg) variants, confirming a diagnosis of Gilbert syndrome. Recognition of this underlying condition prevented further unnecessary cessation of anti-tuberculosis therapy. The patient subsequently resumed an individualized regimen guided by drug susceptibility testing and demonstrated radiological improvement during follow-up.

**Conclusion:**

This case highlights an important diagnostic pitfall during tuberculosis treatment. Persistent indirect hyperbilirubinemia with normal transaminases should prompt consideration of Gilbert syndrome rather than DILI. Early recognition and appropriate genetic testing may prevent unwarranted treatment interruption, reduce the risk of disease progression, and improve treatment continuity. To our knowledge, reports describing Gilbert syndrome masquerading as recurrent suspected DILI during tuberculosis treatment remain exceedingly rare.

## 1 Introduction

Pulmonary tuberculosis is one of the major infectious diseases worldwide (1). In Asian populations, drug-induced liver injury (DILI) associated with anti-tuberculosis medications is relatively common (2); therefore, when abnormalities in liver function occur during treatment, clinicians often first attribute them to DILI. Drug-induced liver injury represents one of the most common adverse events associated with anti-tuberculosis therapy and remains a major cause of treatment interruption, regimen modification, and reduced treatment adherence. Such attribution is clinically pragmatic but may be misleading when the biochemical pattern deviates from classical hepatocellular injury. However, isolated elevations in bilirubin—particularly indirect bilirubin—with normal transaminase levels do not align with the typical biochemical pattern of DILI (3, 4), and represent a diagnostic challenge during tuberculosis treatment.

Gilbert syndrome is an inherited disorder of bilirubin metabolism characterized by intermittent unconjugated hyperbilirubinemia resulting from pathogenic variants in the *UGT1A1* gene (5, 6).The disorder results from reduced activity of uridine diphosphate glucuronosyltransferase 1A1 (*UGT1A1*), leading to impaired bilirubin conjugation and recurrent unconjugated hyperbilirubinemia. Although not rare globally, its prevalence varies notably among ethnic groups. Epidemiological studies report a prevalence of approximately 9–14% in Western populations and 4.3–6.5% in East Asian populations (6). Affected individuals commonly exhibit intermittent jaundice, while liver enzyme levels generally remain normal (4).

Despite its overall prevalence, Gilbert syndrome is rarely recognized in the context of tuberculosis treatment, and the coexistence of Gilbert syndrome and pulmonary tuberculosis is exceedingly uncommon. Only a limited number of cases have been reported in the literature (7, 8), reflecting limited clinical awareness. In the present case, the patient experienced repeated interruptions of anti-tuberculosis therapy due to recurrent hyperbilirubinemia, which was repeatedly misinterpreted as DILI, resulting in a delay in treatment lasting more than 1 year. She was ultimately diagnosed with Gilbert syndrome through *UGT1A1* genetic testing (9). Through this report, we aim to emphasize that, in tuberculosis management, atypical patterns of “liver injury” —particularly isolated indirect hyperbilirubinemia with normal transaminases—should prompt consideration of alternative diagnoses such as Gilbert syndrome, in order to prevent misdiagnosis, unnecessary treatment interruption, and disease progression (1).

## 2 Clinical timeline

The major diagnostic, therapeutic, and follow-up events are summarized in Table 1.

**Table 1:** Clinical timeline of diagnosis and treatment.

Date	Clinical event
Jan 2024	Presented with fever, cough, and sputum production; chest CT revealed right upper lobe lesions suggestive of pulmonary tuberculosis
Jan 2024	Positive interferon-gamma release assay (IGRA); sputum Xpert MTB/RIF negative; bronchoalveolar lavage fluid (BALF) TB-LAMP negative
Jan 2024	Clinically diagnosed with smear-negative, Xpert-negative pulmonary tuberculosis; treatment with isoniazid, rifapentine, ethambutol, and amikacin (HRptEAm) was initiated
Jan–Feb 2024	Rash, fever, and mild hyperbilirubinemia developed; anti-tuberculosis therapy was interrupted because of suspected drug-induced liver injury (DILI)
Sep 2024	Treatment regimen was modified to linezolid, amikacin, isoniazid, and sodium para-aminosalicylate (LZDAHPAS) because of recurrent hyperbilirubinemia
Oct 2024	Marked elevation of ALT and AST (260.1 U/L and 124.4 U/L, respectively); anti-tuberculosis drugs were discontinued and hepatoprotective therapy was initiated
Jan 2025	Anti-tuberculosis therapy was resumed with linezolid, isoniazid, and levofloxacin (LZDHLFX); bilateral lower-limb numbness developed during treatment
Mar 2025	Recurrent hyperbilirubinemia resulted in another treatment interruption
Jun 2025	Follow-up chest CT demonstrated disease progression with cavity formation in the right upper lobe
20 Jun 2025	BALF Xpert MTB/RIF positive, indicating rifampicin-sensitive *Mycobacterium tuberculosis*
Jul 2025	BALF culture positive for *Mycobacterium tuberculosis*, providing microbiological confirmation of pulmonary tuberculosis
Aug 2025	Drug susceptibility testing was completed, followed by *UGT1A1* genetic testing because of persistent isolated unconjugated hyperbilirubinemia
Sep 2025	Gilbert syndrome confirmed by identification of *UGT1A1* variants c.-41_-40dupTA and c.211G>A (p.Gly71Arg)
Oct 2025	Individualized anti-tuberculosis therapy continued; bilirubin levels remained stable and follow-up chest CT demonstrated reduction of the pulmonary cavity

## 3 Case presentation

A 22-year-old woman was admitted with a 1-month history of fever, cough, and sputum production. She had previously been healthy, with no history of chronic disease, and denied smoking, alcohol consumption, or other unhealthy habits. Her family history was unremarkable. Ten days prior to admission, she developed unexplained fever (maximum temperature 38.3 °C), accompanied by chills, mild shortness of breath, a shallow cough, and a small amount of yellow-white mucoid sputum. She reported no headache, dizziness, nausea, vomiting, abdominal pain, or diarrhea. Her appetite had decreased, while bowel and urinary habits remained normal.

On admission, her vital signs were stable, with a temperature of 36.0 °C, pulse rate of 82 beats/min, respiratory rate of 18 breaths/min, and blood pressure of 99/65 mmHg. She was alert and in acceptable general condition. No jaundice was observed on the skin or sclera. The thorax was symmetrical, breath sounds were coarse bilaterally without obvious rales, and cardiac auscultation revealed no murmurs.

**Figure 1:**
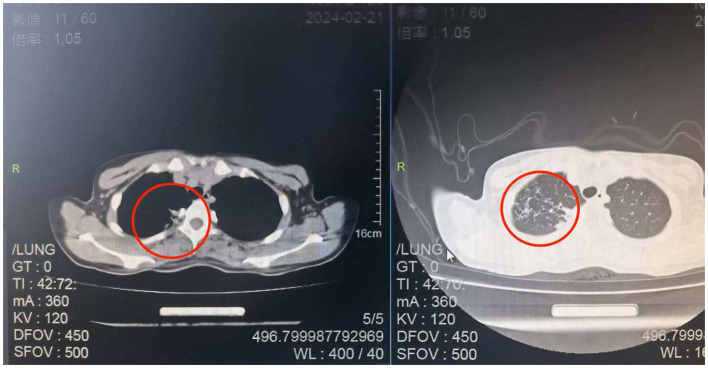
Chest CT before treatment showing patchy and nodular opacities in the right upper lobe.

Chest computed tomography performed on 14 January 2024 demonstrated multiple patchy, cord-like, and nodular areas of increased density in the right upper lobe (Figure 1). Complete blood count and coagulation profile were within normal ranges. Liver and renal function tests were normal except for mildly elevated bilirubin levels: total bilirubin 20 μmol/L, direct bilirubin 6.3 μmol/L, and indirect bilirubin 14.4 μmol/L; total bile acids were 4.8 μmol/L. Serological tests for hepatitis A, B, and C viruses, as well as HIV, were negative. Interferon-gamma release assay was positive, suggesting tuberculosis infection. However, sputum Xpert MTB/RIF testing and drug-resistance gene assays were negative, and the TB-LAMP assay was also negative. Fiberoptic bronchoscopy revealed no abnormalities of the airway mucosa, and lavage fluid TB-LAMP testing was negative. Abdominal ultrasonography showed no abnormalities.

Based on persistent respiratory symptoms, characteristic radiological abnormalities in the right upper lobe, a positive interferon-gamma release assay (IGRA), exclusion of alternative diagnoses, and the diagnostic criteria for smear-negative pulmonary tuberculosis according to Chinese national tuberculosis guidelines (10), the patient was clinically diagnosed with smear-negative, Xpert-negative pulmonary tuberculosis and anti-tuberculosis therapy was initiated. Although initial sputum Xpert MTB/RIF and TB-LAMP assays, as well as bronchoalveolar lavage fluid TB-LAMP testing, were negative, subsequent bronchoalveolar lavage Xpert MTB/RIF positivity and culture confirmation provided microbiological evidence supporting the initial diagnosis. Apart from mildly elevated bilirubin at baseline, all other liver function parameters were within normal limits (Table 2). Given the known hepatotoxic potential of anti-tuberculosis medications, the elevated bilirubin level was initially regarded as a possible early manifestation of drug-induced liver injury.

**Table 2:** Key changes in liver function during anti-tuberculosis treatment.

Test date	ALT (U/L)	AST (U/L)	TBIL (μmol/L)	DBIL (μmol/L)	IBIL (μmol/L)	Total bile acids (μmol/L)
2024-01-25 (baseline)	Within normal range	Within normal range	20.0	6.3	14.4	4.8
2024-09-20 (recurrent hyperbilirubinemia)	Within normal range	Within normal range	35.4	8.7	26.7	6.0
2024-10-23 (suspected DILI episode)	260.1	124.4	24.9	7.4	17.5	4.5
2025-01-09 (after regimen modification)	Within normal range	Within normal range	40.6	11.1	29.5	5.8
2025-07-01 (peak bilirubin fluctuation)	Within normal range	Within normal range	62.3	13.6	48.7	14.2
2025-10-12 (post-adjustment follow-up)	Within normal range	Within normal range	49.6	13.7	35.9	3.4

ALT, alanine aminotransferase; AST, aspartate aminotransferase; TBIL, total bilirubin; DBIL, direct bilirubin; IBIL, indirect bilirubin.

Anti-tuberculosis therapy was initiated with the HRptEAm regimen in January 2024. During treatment, the patient developed a rash and fever accompanied by mild elevations in bilirubin, prompting discontinuation of therapy. Throughout this phase, alanine aminotransferase and aspartate aminotransferase levels remained within normal ranges, with isolated hyperbilirubinemia being the predominant abnormal finding.

From September 2024 to January 2025, the treatment regimen was modified to LZDAHPAS. On 23 October 2024, alanine aminotransferase and aspartate aminotransferase levels increased markedly, while bilirubin levels showed little change. Anti-tuberculosis drugs were again discontinued and hepatoprotective therapy was initiated. On 9 January 2025, after switching to LZDHLFX, the patient developed bilateral lower-limb numbness. In March 2025, bilirubin levels rose once more, leading to another interruption of treatment. During this period, sputum cultures and bronchoalveolar lavage fluid cultures remained persistently negative.

In June 2025, follow-up chest CT demonstrated radiological progression of the right upper lobe lesions with cavity formation. On 20 June 2025, bronchoalveolar lavage Xpert MTB/RIF testing became positive for *Mycobacterium tuberculosis*, with rifampicin susceptibility. In July 2025, bronchoalveolar lavage culture yielded *M. tuberculosis*. Drug susceptibility testing performed on 21 August 2025 revealed resistance to amikacin, kanamycin, and levofloxacin, with susceptibility to other tested agents. From 2024 until June 2025, repeated smear microscopy, Xpert MTB/RIF, and mycobacterial culture examinations remained negative, and definitive microbiological confirmation was delayed. This prolonged diagnostic and therapeutic delay was closely associated with repeated treatment interruptions due to presumed drug-induced liver injury.

### 3.1 Diagnostic assessment

Given the recurrent episodes of hyperbilirubinemia during anti-tuberculosis treatment, several differential diagnoses were considered, including drug-induced liver injury, viral hepatitis, biliary tract disease, hemolytic disorders, and inherited disorders of bilirubin metabolism. Serological testing for hepatitis viruses was negative, and abdominal ultrasonography revealed no evidence of hepatobiliary abnormalities. No clinical or laboratory findings suggested hemolysis. Data regarding hepatitis E virus, Epstein–Barr virus, cytomegalovirus, autoimmune hepatitis, and the use of herbal products or over-the-counter medications were unavailable because these evaluations were not routinely performed during the patient's clinical management. Importantly, bilirubin elevation was predominantly indirect, while alanine aminotransferase and aspartate aminotransferase levels remained persistently normal except during one transient episode. Furthermore, bilirubin levels continued to fluctuate despite discontinuation of anti-tuberculosis drugs, a pattern inconsistent with typical drug-induced liver injury. Because isolated unconjugated hyperbilirubinemia without persistent transaminase elevation is not a characteristic biochemical pattern of anti-tuberculosis drug-induced liver injury, the overall clinical presentation was considered more suggestive of an inherited disorder of bilirubin metabolism. Although a formal RUCAM score was not retrospectively calculated, the persistent bilirubin fluctuations despite drug withdrawal and the absence of sustained hepatocellular injury argued against anti-tuberculosis DILI as the primary explanation. These findings prompted further evaluation for hereditary disorders of bilirubin metabolism.

### 3.2 Diagnosis

*UGT1A1* genetic testing was performed in August 2025 (Table 3). Sanger sequencing subsequently confirmed the c.211G>A variant (Table 4). Results obtained in September 2025 revealed two heterozygous variants: a promoter TA-repeat expansion (c.-41_-40dupTA) and a missense variant (c.211G>A, p.Gly71Arg). Both variants have previously been associated with reduced *UGT1A1* enzymatic activity and unconjugated hyperbilirubinemia. In combination with the persistent pattern of isolated indirect hyperbilirubinemia and consistently normal transaminase levels, the patient was ultimately diagnosed with Gilbert syndrome.

**Table 3:** *UGT1A1* variant interpretation.

Gene	Variant	Protein change	Zygosity	Clinical relevance
*UGT1A1*	c.-41_-40dupTA	—	Heterozygous	Associated with Gilbert syndrome
*UGT1A1*	c.211G>A	p.Gly71Arg	Heterozygous	Associated with Gilbert syndrome; confirmed by Sanger sequencing

*UGT1A1*, UDP-glucuronosyltransferase 1A1. Both variants have been previously reported in association with Gilbert syndrome and supported the final diagnosis in this patient.

**Table 4:** Sanger sequencing confirmation of the *UGT1A1* c.211G>A variant.

Locus information		Relationship	Sample ID	validation result	
*UGT1A1* chr2:234669144, Exon 1/5		Proband	NK27D01010	Heterozygous	
NM_000463.3:c.211G>A (p.Gly71Arg)					
Chromatogram findings					
Gene	*UGT1A1*	Genomic Position	chr2:234669144	Variant Information	c.211G>A (p.Gly71Arg)
NK27D01010 Proband forward sequencing Heterozygous		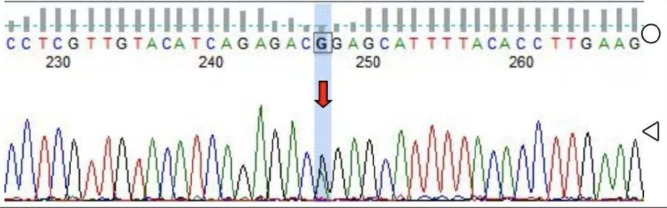			

### 3.3 Therapeutic Intervention, Follow-up and Outcomes

**Figure 2:**
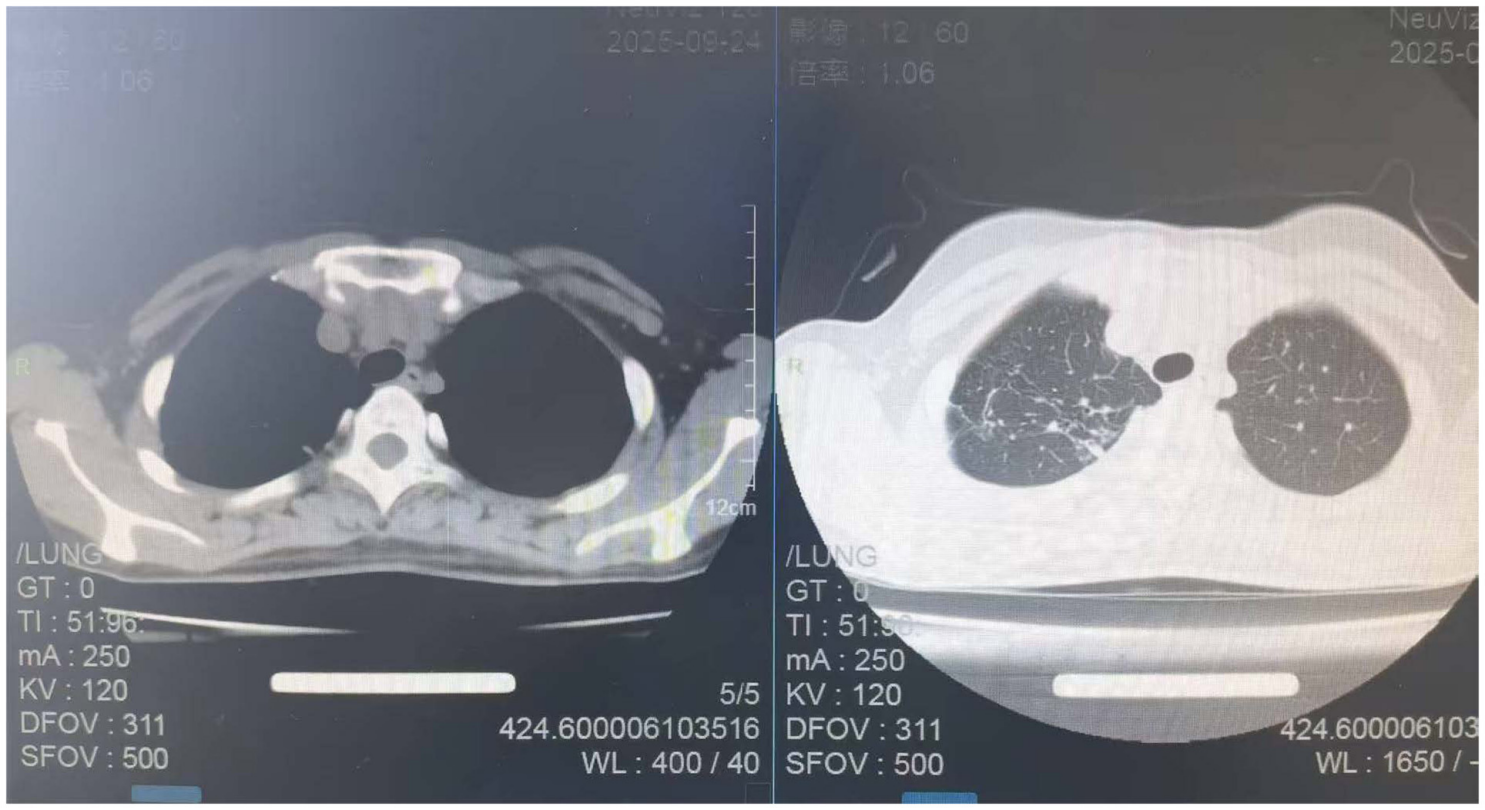
Follow-up chest CT demonstrating reduction in cavity size after adjusted anti-tuberculosis therapy.

After adjustment of the anti-tuberculosis regimen according to drug susceptibility results, the patient continued treatment under close monitoring. Mild fluctuations in bilirubin levels persisted, but overall values remained stable, and no evidence of hepatocellular injury was observed. Follow-up chest CT demonstrated a reduction in the size of the right upper lobe cavity (Figure 2), indicating radiological improvement after continuation of individualized anti-tuberculosis therapy.

## 4 Discussion

This patient, undergoing initial treatment for pulmonary tuberculosis, experienced repeated episodes of “liver function abnormalities” characterized primarily by elevated bilirubin. Because first-line anti-tuberculosis medications—such as isoniazid, rifampicin, and pyrazinamide—are strongly associated with drug-induced liver injury (DILI) (3, 4), she was repeatedly managed as having DILI and her treatment was intermittently discontinued. This clinical decision is understandable in routine tuberculosis practice but may be inappropriate when biochemical features deviate from classical hepatocellular injury. However, the characteristics of her bilirubin abnormalities were inconsistent with typical DILI: (1) elevation was predominantly in indirect bilirubin; (2) ALT and AST remained normal or showed only mild fluctuations; (3) bilirubin did not return to normal after discontinuation of anti-tuberculosis drugs; and (4) she lacked symptoms classically associated with hepatocellular injury, such as significant fatigue or nausea. These features instead suggested a disturbance of bilirubin metabolism rather than drug-induced hepatocellular damage (5).

Subsequent *UGT1A1* genetic testing identified two heterozygous variants—c.-41_-40dupTA and c.211G>A (p.Gly71Arg)—both of which are known pathogenic or pathogenic-associated variants of Gilbert syndrome (9).Previous population studies have also demonstrated that *UGT1A1* polymorphisms are strongly associated with serum bilirubin concentrations and the clinical phenotype of Gilbert syndrome (11), further supporting the pathogenic role of UGT1A1 variants identified in the present patient. As an autosomal recessive disorder, Gilbert syndrome is characterized by mild, recurrent unconjugated hyperbilirubinemia and is not uncommon in the general population. Notably, despite its relatively high prevalence, Gilbert syndrome is rarely considered during tuberculosis treatment, particularly when bilirubin abnormalities arise in the absence of overt transaminase elevation. Nevertheless, coexistence of pulmonary tuberculosis and Gilbert syndrome is exceedingly rare, and clinicians often attribute bilirubin abnormalities solely to DILI, resulting in unnecessary treatment interruptions and delayed tuberculosis management (7, 8).

Previous reports have described both drug-resistant and drug-susceptible tuberculosis patients who developed recurrent hyperbilirubinemia due to underlying Gilbert syndrome, leading to repeated medication changes, treatment pauses, and compromised therapeutic outcomes. The clinical course of this patient similarly reflects this dilemma: failure to recognize the non-hepatocellular nature of the bilirubin abnormality led to repeated interruption of effective anti-tuberculosis therapy, and earlier recognition of Gilbert syndrome through genetic testing could have prevented more than a year of treatment interruption and delay.

Ultimately, microbiological confirmation of tuberculosis was achieved through positive Xpert testing and culture of bronchoalveolar lavage (12). The delayed microbiological confirmation and radiological progression observed in this case underscore the clinical consequences of unnecessary treatment cessation. This highlights the importance of carefully evaluating abnormal laboratory findings during tuberculosis treatment while ensuring appropriate disease control. When bilirubin elevation occurs in the absence of transaminase elevation or evidence of hepatocellular injury, Gilbert syndrome should be actively included in the differential diagnosis rather than regarded as an exclusion diagnosis after repeated treatment failure. Under close monitoring, anti-tuberculosis therapy should be continued to minimize the risk of disease progression caused by unnecessary treatment cessation.

In summary, this case emphasizes that isolated indirect hyperbilirubinemia during anti-tuberculosis therapy should raise a strong suspicion for Gilbert syndrome (5, 6), prompting early *UGT1A1* genetic testing. From a tuberculosis management perspective, recognition of this diagnostic pitfall is essential to balancing drug safety with treatment continuity. Such an approach not only helps avoid misdiagnosis and unwarranted treatment interruption but also ensures the uninterrupted completion of the anti-tuberculosis regimen, thereby improving therapeutic success.

A limitation of this report is that it describes a single patient, which limits the generalizability of the findings. Nevertheless, the case highlights an important diagnostic pitfall that may be encountered in routine tuberculosis management and underscores the value of genetic testing in selected patients with atypical biochemical profiles.

This case also highlights the potential value of precision medicine approaches in tuberculosis management, where genetic testing may help distinguish inherited metabolic conditions from treatment-related adverse events and support individualized clinical decision-making (13).

### 4.2 Clinical lessons

The following clinical lessons can be drawn from this case:

1. Isolated indirect hyperbilirubinemia during anti-tuberculosis therapy should prompt consideration of Gilbert syndrome rather than drug-induced liver injury.
2. Normal or minimally fluctuating transaminases argue against classical hepatocellular injury and should trigger evaluation for alternative causes.
3. Early recognition of atypical biochemical patterns and timely *UGT1A1* genetic testing enables accurate diagnosis, prevents unnecessary treatment interruption, and reduces the risk of tuberculosis progression.
4. Persistent bilirubin elevation despite drug withdrawal is an important clue suggesting a non-hepatic etiology.

### 4.3 Patient perspective

The patient reported considerable anxiety and frustration during the prolonged treatment course because anti-tuberculosis therapy was repeatedly interrupted due to suspected drug-induced liver injury. She found it difficult to understand why bilirubin abnormalities persisted despite discontinuation of medication. After receiving a definitive diagnosis of Gilbert syndrome through genetic testing, she expressed relief at finally having an explanation for the recurrent laboratory abnormalities. The patient was reassured regarding the benign nature of Gilbert syndrome and was willing to continue anti-tuberculosis therapy under close monitoring.

## 5 Conclusion

This case highlights that when “liver function abnormalities” arise during anti-tuberculosis therapy—specifically, isolated indirect hyperbilirubinemia with normal or only mildly fluctuating transaminases—these findings should not be attributed solely to drug-induced liver injury. Failure to recognize this distinction may lead to repeated treatment interruption and compromise tuberculosis control. The possibility of Gilbert syndrome should be considered concurrently. *UGT1A1* genetic testing enables rapid, definitive diagnosis, preventing unnecessary treatment interruptions and delays. Early recognition and individualized treatment planning are crucial to ensuring treatment continuity and optimizing therapeutic outcomes in tuberculosis management.

## Data Availability

The original contributions presented in the study are included in the article/supplementary material, further inquiries can be directed to the corresponding author.
